# Radiological and surgical aspects of polymorphous low-grade neuroepithelial tumor of the young (PLNTY)

**DOI:** 10.1007/s13760-023-02231-z

**Published:** 2023-03-10

**Authors:** Daniele Armocida, Luigi Valentino Berra, Alessandro Frati, Antonio Santoro

**Affiliations:** 1 A.U.O. “Policlinico Umberto I”, Neurosurgery Division, Sapienza University, Rome, Italy; 2 Human Neurosciences Department, Via del Policlinico, 155, 00161 Rome, Italy

## Abstract

**Background:**

Polymorphous low-grade neuroepithelial tumor of the young (PLNTY) is a low-grade epilepsy-associated tumor recently introduced in WHO 2021 classification. Since it has been recognized as an independent nosological entity, PLNTY has been mainly studied from a genetic and molecular perspective, not recognizing unique characteristic clinical and radiological features.

**Methods:**

A systematic literature research has been conducted aiming to identify all relevant studies about the radiological, clinical and surgical features of PLNTY. We described a representative case of a 45-year-old man treated with awake-surgery with confirmed diagnosis of PLNTY, reporting the radiological and surgical characteristics through imaging and intra-operative video. We performed a statistical meta-analysis attempting to assess the presence of relationships between surgical and radiologic tumor characteristics and clinical outcome and type of surgery.

**Results:**

A total of 16 studies were included in the systematic review. The final cohort was composed of 51 patients. Extent of resection (EOR) and outcome are not significantly associated with the different genetic profiling (*p* = 1), the presence of cystic intralesional component, calcification (*p* = 0.85), contrast-enhancing and lesion boundaries (*p* = 0.82). No significant correlation there is between EOR and remission or better control of epilepsy-related symptoms (*p* = 0.38). The contrast enhancement in the tumor is significantly associated with recurrence or poor control of epileptic symptoms (*p* = 0.07).

**Conclusions:**

In PLNTYs, contrast enhancement seems to impact prognosis, recurrence, and seizure control much more than radiological features, genetic features and type of resection of the tumor.

**Supplementary Information:**

The online version contains supplementary material available at 10.1007/s13760-023-02231-z.

## Introduction

Polymorphous low-grade neuroepithelial tumor of the young (PLNTY) is a low-grade tumor comprised of infiltrating “oligodendrocyte-like” cells [1] that stain with CD34 immunopositivity [2]. It frequently affects children and young adults, with a slightly female predominance [3]. PLNTY is a typical low-grade epilepsy-associated tumor recently described and introduced in Central Nervous Tumor-World Health Organization (CNS-WHO) 2021 classification [2, 3]. Before the last updated classification, PLNTY it was considered to take part of a group known as low-grade epilepsy-associated tumors (LEATs), an umbrella of developmental tumors, with favorable clinical outcomes, with varying histologic appearances, associated with early-onset seizures, since other glial tumors, such as pilocytic astrocytomas or oligodendrogliomas, usually had distinct diagnostic criterion that assists in further clinical management [1, 4]. Several distinguishing factors of PLNTY may be implicated in its pathogenesis and clinical manifestation, the most suspicious of which are BRAF V600E gene mutant, increased CD34 expression, and FGFR gene fusion products [5].

Radiologically, clinically and surgically still, very little is known about this rare tumor. The diagnostic challenge most often involves distinguishing this tumor from an oligodendroglioma, given, especially in adults, the morphologic and clinical overlap and the potential for misdiagnosis are significant [1]. Because of their rare etiology, only a few PLNTYs have been described to date. It is unclear how its characteristics impact the pathogenesis and the outcome of the disease [6]. Clinically, Oligodendroglioma is associated with a lower rate of epilepsy and a worse clinical prognosis. At the same time, PLNTYs, tend to exhibit a benign (typical of WHO grade I) clinical course, and appear to be well controlled by gross total resection (GTR). However, some reports describe cases of difficult therapeutic control or disease recurrence [7–9].

PLNTYs, indeed, can be morphologically highly variable, manifesting as well-circumscribed lesions, cystic lesions, or infiltrative growth patterns. The constant element appears to be the calcific component that is always present in the context of the lesion, leading it to be in differential diagnosis with oligodendroglioma [1]. In this review and meta-analysis, we want to identify the clinical, radiological, and surgical features known about PLNTY, reporting a typical case of misdiagnosis with adult oligodendroglioma from our experience, providing as much surgical information as possible through the presentation of an intra-operative video. In addition, by reviewing the research on cases reported in other series, we tried to conclude that the radiological characteristics and genetic alterations of PLNTY could predict the outcome of this new kind of epileptogenic tumor.

## Methods

An extensive systematic search has been conducted on Pubmed according to the Preferred Reporting Items for Systematic Reviews and Meta-analysis (PRISMA) guidelines, to identify all potentially relevant studies about the radiological, clinical, and surgical characteristics of PLNTY reported. In searching for relevant studies, the reference section of included articles was analyzed. The search was performed by typing the following items: “Polymorphous low-grade neuroepithelial tumor of the young AND/OR PLNTY.”

The first selection step was describing the clinical entity of PLNTY in pediatric and adult patients and its characteristics.

In this regard, we chose to evaluate only the studies focused on PLNTY only PLNTY-focused studies and papers that described patients or series reported and described clinically and radiologically.

Given these premises, we selected papers according to the following inclusion criteria:

- Availability of full-text articles;
- English text only;
- Patients with a diagnosis of PLNTY;
- Reported details on imaging;
- Presence of neurological outcome evaluation. No specific limitation was applied regarding the timing of neurological evaluation after treatment.

Conversely, exclusion criteria were:

- Full-text articles in languages other than English;
- Studies reporting patients with the low-grade tumors;
- No data available about the neurological outcome;
- No imaging details were reported;

Data extracted from each study were (1) authors, (2) year of publication, (3) number of patients included, (4) age, (5) sex, (6) clinical debut and presentation on diagnosis, (7) side involved, (8) major cerebral lobe involved, (9) imaging characteristics, (10) presence of contrast-enhancing in magnetic resonance imaging (MRI) studies, (11) major diameter of tumor in MRI studies, (12) characteristics of radiological limits of the lesion (defined as “circumscribed” when limits are well-identifiable on MRI, in contrast on “blurred” limits), (13) mutation identified on diagnostic molecular analysis, (14) surgical treatment indicating the extent or resection (EOR), (15) clinical and neurological outcome after treatment.

We added and described a representative case from our experience reporting the radiological and surgical characteristics through imaging and intra-operative video. From the available data, we performed a statistical meta-analysis attempting to assess the presence of significant relationships between surgical and radiologic tumor characteristics and clinical outcome and type of surgery.

## Case description

A 45-year-old right-handed man without relevant clinical history presented to our institution for partial seizure in left hand. In addition, He reported approximately two recent episodes of extreme fatigue in which he would fall asleep and be unarousable for hours, as well as shorter duration facial and arm paresthesias. The patient was admitted for neurological care and underwent neuroimaging to rule out a structural cause for his symptoms. The patient underwent a preoperative brain MRI scan included a high-field 3 Tesla volumetric study with the following sequences: T2w, FLAIR, isotropic volumetric T1-weighted magnetization-prepared rapid acquisition gradient echo (MPRAGE) before and after intravenous administration of paramagnetic contrast agent; diffusion tensor sequences (DTI) with 3D tractography and functional MRI (fMRI). The procedure was performed with an infrared-based Neuronavigator (Brainlab, Kick^®^ Purely Navigation), in a standard neurosurgical theatre, with a standard operative microscope (Leica, model OH4). In the first postoperative day, as routine, the patients underwent volumetric Brain MRI scan to evaluate the EOR.

### Imaging

An initial non-contrast head CT showed a partially calcified intra-axial mass in the right temporoparietal lobe. MR imaging confirmed the presence of the lesion in the peritrigonal area involving the corpus callosum and internal capsule, which demonstrated a heterogeneous signal with few intralesional calcifications. Faint T1 hyperintensity was seen within the tumor, while other regions demonstrated minimal enhancement. Focal regions of elevated relative CBV were noted within the tumor on DSC perfusion imaging. A mild associated mass effect was seen, with effacement of the adjacent right lateral ventricle and expansion of an overlying right temporal lobe gyrus, DTI show just a minimal displacement of with fibers involving the right arcuate fasciculus. However, there was no midline shift or herniation. Preoperative imaging was completed on a 3T MR imaging scanner to optimize fMRI and spectroscopy. On this examination, the signal with multivoxel did not show significant spikes or reversal of the choline/*n*-acetyl-aspartate (Cho-NAA) ratio. Initial imaging raised concern for an oligodendroglioma, though location and dense central calcifications were thought to be atypical.

**Fig. 1:**
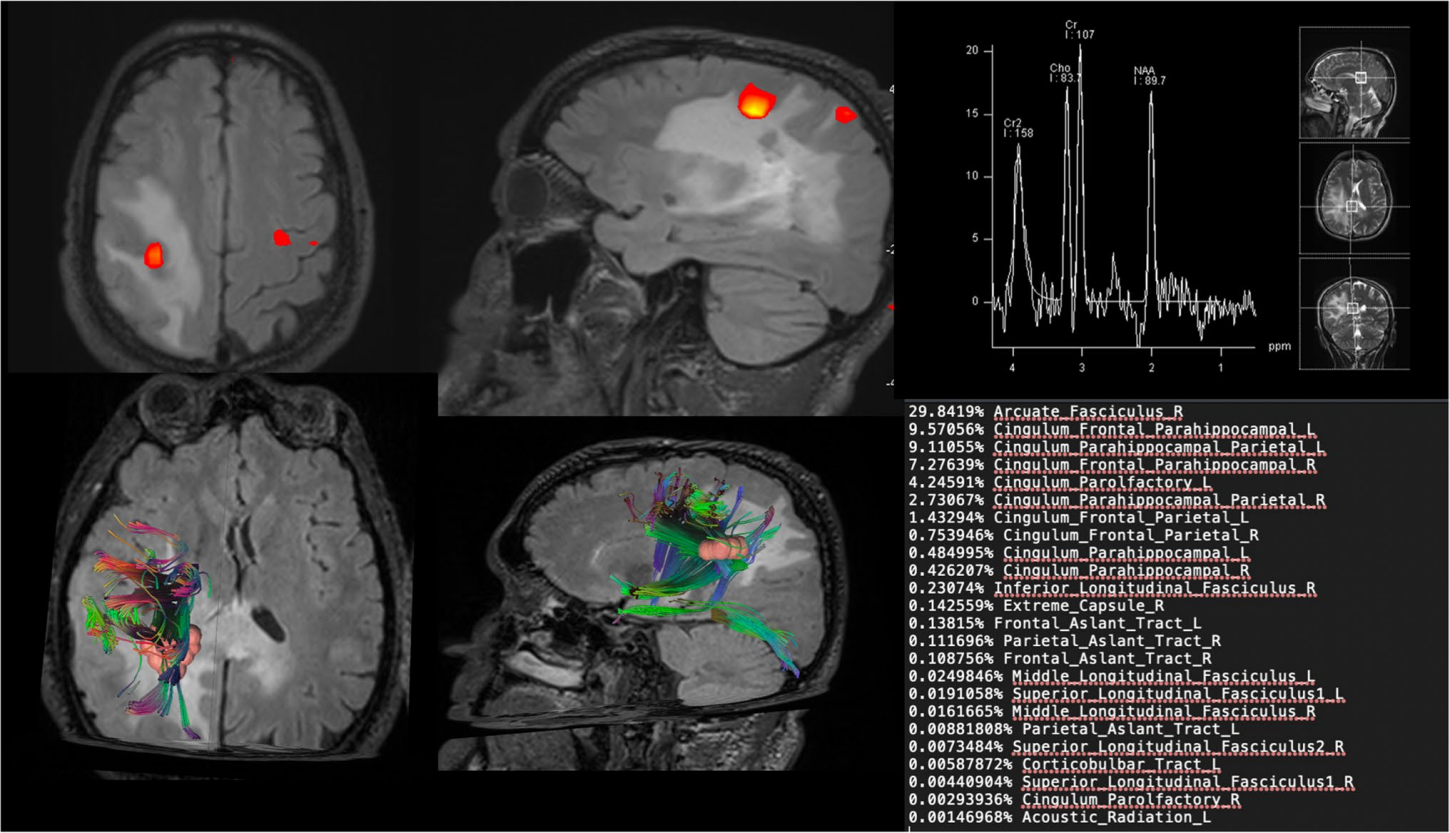
The panel shows the MRI images obtained in our representative case documenting the presence of the unimpaired motor area of the hand in its physiological position. The DTI images document little infiltration of the tumor mass with partial involvement of the arcuate fascicle of the right hemisphere. Spectroscopy examination shows a minimal choline peak without inversion only in the deep peri-callosal part of the mass

Preoperative language fMRI was predominantly used to establish the patient’s hemispheric language dominance and the dislocation of the primary motor area; because the tumor location and posterior corpus callosum involvement would have necessitated an awake resection with electrocortical stimulation mapping to prevent a postoperative motor and coordination deficit. The patient performed fMRI language tasks in the scanner. The derived statistical maps demonstrated left-hemispheric language dominance (Fig. 1).

The patient underwent resection of the mass via a right temporoparietal craniotomy. The surgeons elected to approach the tumor through a corticectomy of the right superior parietal lobule, the shortest route to the tumor given the reassuring fMRI data. Intraoperative evaluation of the tumor demonstrated a high variable lesion in consistency, aspect, and coloration (Video 1). The tumor was just partially removed in a piecemeal fashion. The debulking was stopped in case of speech or motor arrest of patients. In the postoperative phase, the patient manifested hemiparesis in the left arm and recovered over the next 2 weeks after the procedure. No manifestations of tactile sensory alterations, stereoagnostic disturbances, or visuospatial localization have been reported.

The patient did well postoperatively without immediate complications. Histological diagnosis confirmed the presence of PLNTY (grade I WHO) with ki-67 = 3% and BRAF-V600E mutation.

### Statistical methods

The sample was analyzed with SPSS version 18 (SPSS Inc., Chicago, Illinois, USA). A comparison between nominal variables was made with the chi-square test. Means of continuous variables were compared with one-way and multivariate analysis of variance along with contrast analysis and post hoc tests. Continuous variable correlations were investigated with Pearson bivariate correlation. The threshold of statistical significance was considered at *p* < 0.05.

## Results

**Fig. 2:**
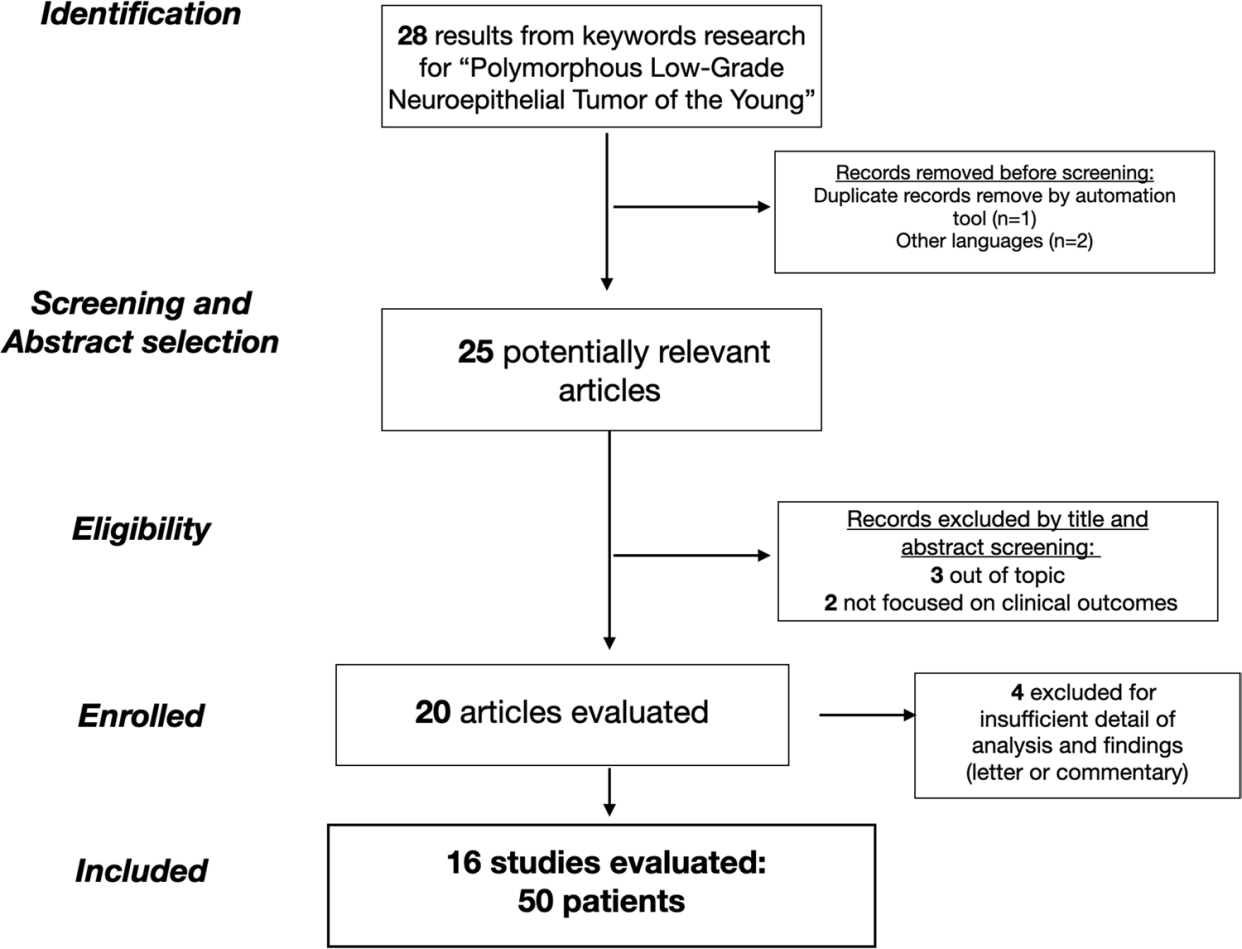
Selection study flowchart

A total of 28 studies were found through PubMed database search and reference section screening. An automatic tool working on Microsoft Excel spreadsheets carried out a duplicate check. Out of 28 initial papers, one duplicate and 2 paper with another language other than English were removed; thus 25 potentially articles were identified, and, following the eligibility criteria, 20 papers were screened. In particular, manuscripts did not fulfilling the eligibility criteria, because they were out of topic (focusing on general low-grade tumor of low-grade CD34+, three papers), not focusing on clinical characteristics (genomic or biological analysis, two papers), and four papers were excluded for insufficient detail of case analysis and finding (four papers). A total of 16 articles were included in the systematic review. Out of 50 patients overall included, 1 patient was successfully added to the analysis from our institutional experience. The selection flowchart diagram is reported in Fig. 2. Due to the low number of patients in the final series, patient demographics, clinical data, and tumor imaging characteristics are reported descriptively in Table 1.

**Table 1:** Reported cases of PLNTY in the literature

No.	Authors	Year	Pts	Age	Sex	Clinical presentation	Side	Major lobe involvement	Imaging	Contrast enhancement	Major diameter (mm)	Limits of lesion	Mutation	Treatment	Outcome
1	Bale et al. [7]	2021	1	15	F	Seizure	Left	Temporal	Calcified, cystic	Yes	12	Circumscribed	FGFR3-TACC3	GTR	Shift, recurrence
2	Bitar et al. [8]	2018	1	31	M	Epilepsy	Right	Temporal	–		–	Blurred	BRAF V600E	Lobectomy	Re-surgery
3	Chen et al. [14]	2020	3	14	F	Seizure	Left	Temporal	Calcified, cystic	No	–	Blurred	FGFR3-TACC3	GTR	Under control
				15	M	Seizure	Right	Temporal	Calcified, cystic	Yes	–	Blurred	BRAF V600E	GTR	Under control
				16	M	Seizure	Right	Frontal	Calcified, cystic	Yes	–	Blurred	N/A	GTR	Under control
4	Gupta et al. [1]	2021	5	11	M	Epilepsy	Right	Frontal	–	–	–	–	FGFR2	–	–
				17	M	Epilepsy	Left	Temporal	–	–	–	–	FGFR2	–	–
				10	F	Epilepsy	Left	Temporal	–	–	–	–	FGFR2	–	–
				38	M	Seizure	Right	Temporal	–	–	–	–	FGFR2	–	–
				11	F	Epilepsy	Right	Temporal	–	–	–	–	FGFR2	–	–
5	Gupta V.R. et al. [2]	2019	1	30	M	Epilepsy	Right	Temporal	Solid	No	6	Circumscribed	BRAF V600E	Lobectomy	Seizure-free
6	Huse et al. [9]	2016	10	16	M	Seizure	Right	Temporal	Calcified	No	7	Circumscribed	BRAF V600E	GTR	
				18	F	Seizure	Right	Temporal	Calcified, cystic	Yes	–	Circumscribed	BRAF V600E	PR	Psychosis
				23	F	Seizure	Right	Temporal	Calcified	No	–	Circumscribed	BRAF V600E	–	–
				17	F	Seizure	Right	Temporal	Calcified	No	–	Circumscribed	FGFR3-TACC3	GTR	Seizure-free
				4	M	Seizure	Left	Temporal	Calcified	No	30	Circumscribed	FGFR2	GTR	Seizure-free
				9	M	Seizure	Right	Frontal	Cystic	Yes	–	Circumscribed	KIAA1598-FGFR2 fusion	GTR	Re-surgery
				10	M	Headache	Right	Occipital	Calcified	No	–	Circumscribed	KIAA1598-FGFR2 fusion	GTR	Under control
				23	F	Seizure	Right	Temporal	Calcified	No	–	Circumscribed	–	GTR	Under control
				32	F	Seizure	Right	Temporal	Calcified	No	–	Circumscribed	–	GTR	Seizure-free
				24	F	Visual disturbances	Right	Occipital	Calcified	No	–	Circumscribed	–	GTR	Re-surgery
7	Jhonson et al. [17]	2019	9	12	F	–	Left	Temporal	Calcified, cystic	No	–	Circumscribed	KIAA1598-FGFR2 fusion	–	–
				12	F	–	Left	Temporal	Calcified, cystic	No	–	Circumscribed	FGFR2	–	–
				26	F	–	Left	Temporal	Calcified, cystic	Yes	–	Circumscribed	BRAF V600E	–	–
				16	F	–	Right	Temporal	Calcified	No	–	Circumscribed	BRAF V600E	–	–
				25	M	–	Right	Temporal	Cystic	No	–	Blurred	BRAF V600E	–	–
				15	F	–	Left	Temporal	Calcified, cystic	No	–	Circumscribed	NTRK2 disruption	–	–
				5	F	–	Right	Parietal	Calcified, cystic	No	–	Circumscribed	KIAA1598-FGFR2 fusion	–	–
				34	M	–	Right	Temporal	Calcified, cystic	Yes	–	Circumscribed	BRAF V600E	–	–
				17	F	–	Median	Third ventricle	Calcified, cystic	Yes	–	Circumscribed	BRAF V600E	–	–
8	Riva et al. [15]	2018	1	57	M	Headache	Right	Frontal	Cystic	No	25	Blurred	FGFR3-TACC3	GTR	Asymptomatic
9	Sumdani et al. [5]	2019	1	19	M	Seizure	Right	Parietal	Calcified	No	15	Blurred	BRAF V600E	GTR	Under control
10	Surrey et al. [18]	2019	6	7	M	Epilepsy	–	Temporal	Cystic	–	–	–	FGFR2	GTR	Under control
				10	F	Epilepsy	–	Parietal	–	–	–	–	FGFR2	GTR	Re-surgery
				14	M	Epilepsy	–	Parietal	–	–	–	–	FGFR3-TACC3	GTR	Seizure-free
				16	M	Epilepsy	–	Temporal	–	–	–	–	BRAF V600E	GTR	Seizure-free
				8	M	Epilepsy	–	Temporal	Cystic	–	–	–	BRAF V600E	GTR	Seizure-free
				14	F	Epilepsy	–	Temporal	Cystic	–	–	–	BRAF V600E	GTR	Seizure-free
11	Tateishi et al. [6]	2020	1	14	M	Epilepsy	Left	Temporal	Calcified, cystic	No	20	Circumscribed	BRAF V600E	GTR	Seizure-free
12	Fei et al. [19]	2022	8	5	M	Epilepsy	Right	Occipital	Cystic	No	–	–	N/A	PR	Seizure-free
				25	M	Seizure	Right	Temporal	Calcified	No	–	–	BRAF V600E	PR	Seizure-free
				21	M	Epilepsy	Right	Temporal	Calcified, cystic	Yes	–	–	BRAF V600E	GTR	Seizure-free
				51	M	Epilepsy	Left	Frontal	Calcified	Yes	–	–	N/A	GTR	Seizure-free
				30	F	Seizure	Right	Frontal	Calcified	No	–	–	N/A	GTR	Seizure-free
				46	M	Epilepsy	Left	Temporal	Calcified, cystic	No	–	–	N/A	GTR	Under control
				28	M	Epilepsy	Left	Temporal	Calcified	No	–	–	BRAF V600E	GTR	Seizure-free
				47	M	Epilepsy	Right	Frontal	Cystic	Yes	–	–	BRAF V600E	GTR	Under control
14	Lelotte et al. [3]	2019	1	33	F	Seizure	Right	Temporal	Cystic	No	25	Blurred	BRAF V600E	GTR	Under control
15	Broggi et al. [	2021	1	50	F	Epilepsy	Left	Temporal	Calcified	No	20	Circumscribed	N/A	GTR	Under control
16	Benson et al. [16]	2021	1	44	F	Mood disorder	Left	Temporal	Calcified, cystic	Yes	–	Circumscribed	BRAF V600E	GTR	Under control
17	Our case	2022	1	45	M	Seizure	Right	Parietal	Calcified	Yes	45	Blurred	BRAF V600E	PR	Under control

### Population analysis

The final cohort was composed of 51 patients. The average age of the patients was 22.16 years (min = 4, max = 57), with a slight predominance of male sex (54.9% versus 45%). The main symptom of the onset is seizure comprehensive of a single seizure attack (18 patients, 42.9%) and intractable epilepsy (20 patients, 47.6%). Other symptoms at onset reported are headache (two patients, 4.8%), visual disturbance (one patient, 2.4%), and mood disorder (one patient with depression, 2.4%).

Regarding the genetic profile, the most expressed mutation is BRAF V600 (23 pts—47.9%) followed by FGFR2 alterations (9 pts—18.8%), FGFR3-TACC3 (5 pts—10.4%), KIAA1598-FGFR2 fusion (4 pts—8.33%), NTRK2 disruption (1 patient—2.1%), although 6 patients do not present a specific alteration (6 pts—12.5%). We analyzed the reported radiological features of cystic intralesional component, calcification and contrast-enhancing and data available about the choice of treatment and clinical outcome. None of the reported cases described the features the tumor took on about the eloquent areas and white matter bundles identifiable on tractography examinations. All the relevant details are included in Table 2.

**Table 2:** Study population

No. cases 51		
Age	Mean 22.16	Min = 4 Max = 57
Sex	M: 28–54.9%	*p* = 1
	F: 23–45%	
Clinical debut (42 pts)	Seizure: 18–42.9%	
	Epilepsy: 20–47.6%	
	Headache: 2–4.8%	
	Visual disturbance: 1–2.4%	
	Mood disorder: 1–2.4%	
Side (45 pts)	Left: 15–33.3%	*p* = 0.26
	Right: 29–64.4%	
	Median: 1–2.2%	
Major lobe involvement (51 pts)	Temporal lobe: 35–68.6%	
	Frontal lobe: 7–13.7%	
	Occipital lobe: 3–5.9%	
	Parietal lobe: 5–9.8%	
	Third ventricle: 1–2%	
Compactness 31 pts	Cystic: 17–54.8%	*p* = 0.39
	No cystis: 14–14–45.16%	
Calcification 42 pts	Calcified: 32–76.2%	*p* = 0.85
	No calcifications: 10–23.8%	
Contrast enhancement 40 pts	Enhancement 13–32.5%	*p* = 0.07
	No contrast enhancing 27–67.5%	
Definition (30 pts)	Circumscribed: 23–76.7%	*p* = 0.34
	Blurred: 9–30%	
Major diameter (10 pts)	Mean: 20	Min = 6 Max = 45
Genetic alteration (48 pts)	BRAF V600 = 23–47.9%	
	FGFR3-TACC3 = 5–10.4%	
	FGFR2 alterations = 9–18.8%	
	KIAA1598-FGFR2 fusion = 4–8.33%	
	NTRK2 disruption = 1–2.1%	
	Not specific alteration = 6–12.5%	
Treatment (35 pts)	GTR = 30–85.7%	0.38
	PR = 4–11.4%	
	Lobectomy = 5.7%	
Outcome (35 pts)	Re-surgery = 5–14.3%	0.78
	Seizure free = 15–42.9%	
	Under control = 13–37.1%	

### Analysis on EOR

The descriptive analysis of the literature shows how important EOR is in treating PLTNY. We analyzed the main clinical and radiological factors that may result in gross total resection of the tumor. We did not identify any significant variables regarding the achievement of complete resection of the tumor. In particular, we analyzed how radiologic features could impact the different types of surgical treatment selected in terms of EOR. Analyzing the presence of cystis, calcification, and lesion boundaries (blurred or circumscribed), we did not find any statistically significant correlation (cystic and calcification related to EOR, *p* = 0.85, lesion boundaries (blurred or circumscribed) with EOR, *p* = 0.82).

### Analysis on outcome

**Fig. 3:**
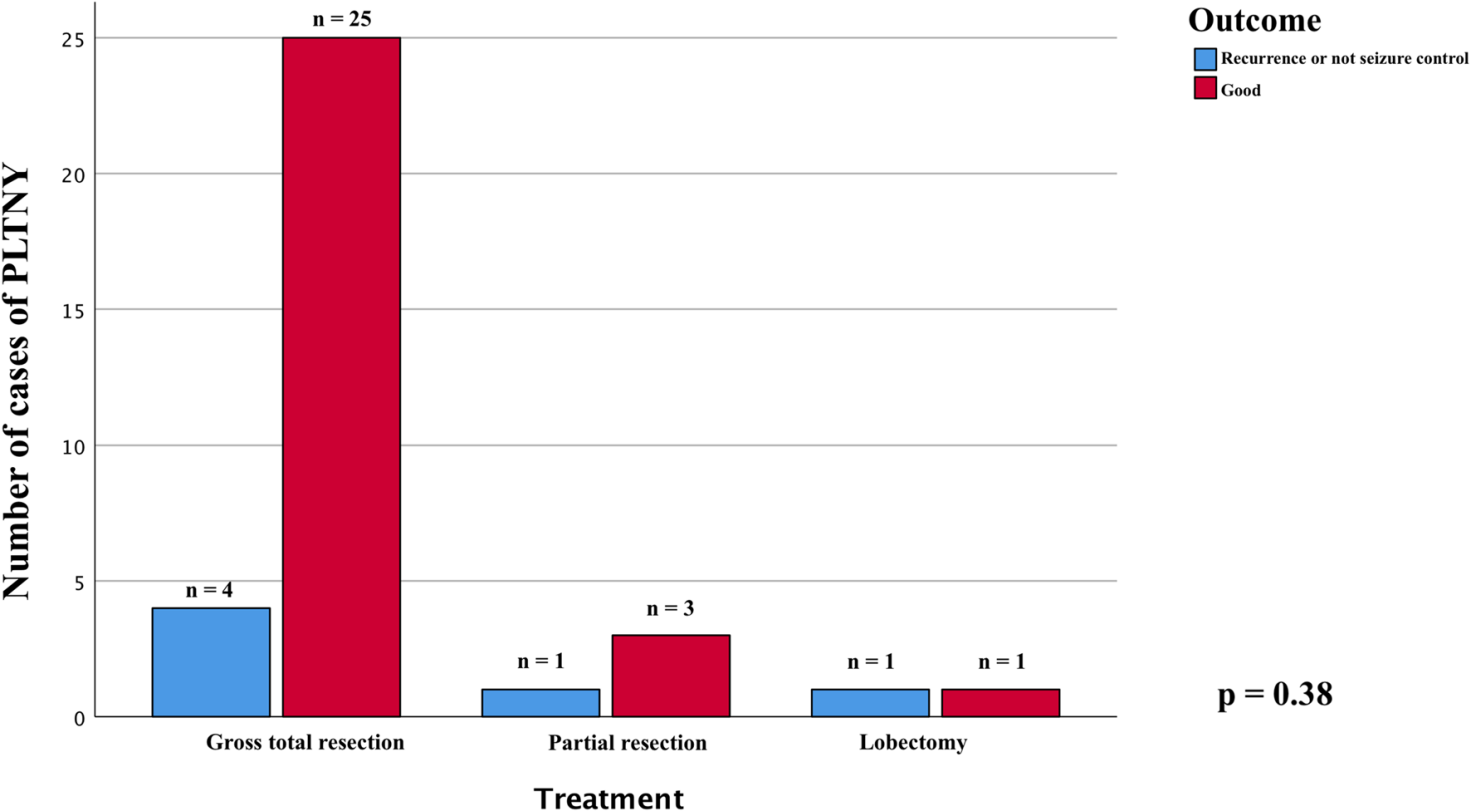
The chi-square analysis displayed graphically in these bar charts shows that the presence of calcifications that is not significantly associated with the outcome of treated patients (PART A *p* = 0.85), while there is a mild significant association between patients with and without contrast-enhancement of the lesion and the outcome (PART B, *p* = 0.07)

We further analyzed the main variables that could, according to our hypothesis, most influence patients' outcomes: we first evaluated how an EOR corresponding to a gross total resection correlated with remission or better control of epilepsy-related symptoms, and it turns out that there is no statistically significant difference (chi-square test *p* = 0.38 Fig. 3), and secondly the principal clinical variables such age, sex, clinical debut and tumor location without any significant association.

**Fig. 4:**
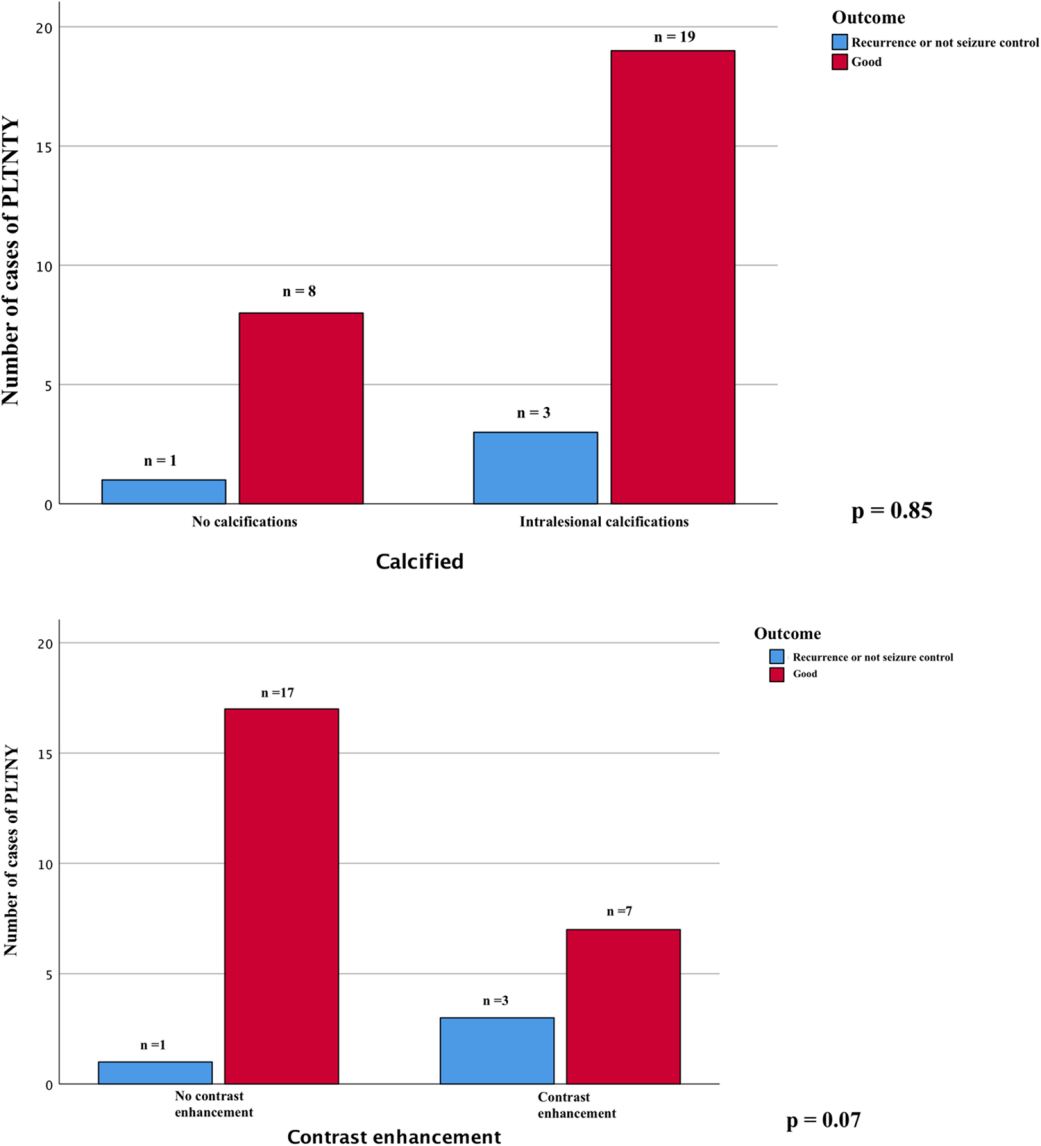
Chi-square comparison analysis shows the different outcomes from the patients treated with gross total resection (GTR), lobectomy, and partial resection (*p* = 0.38)

**Fig. 5:**
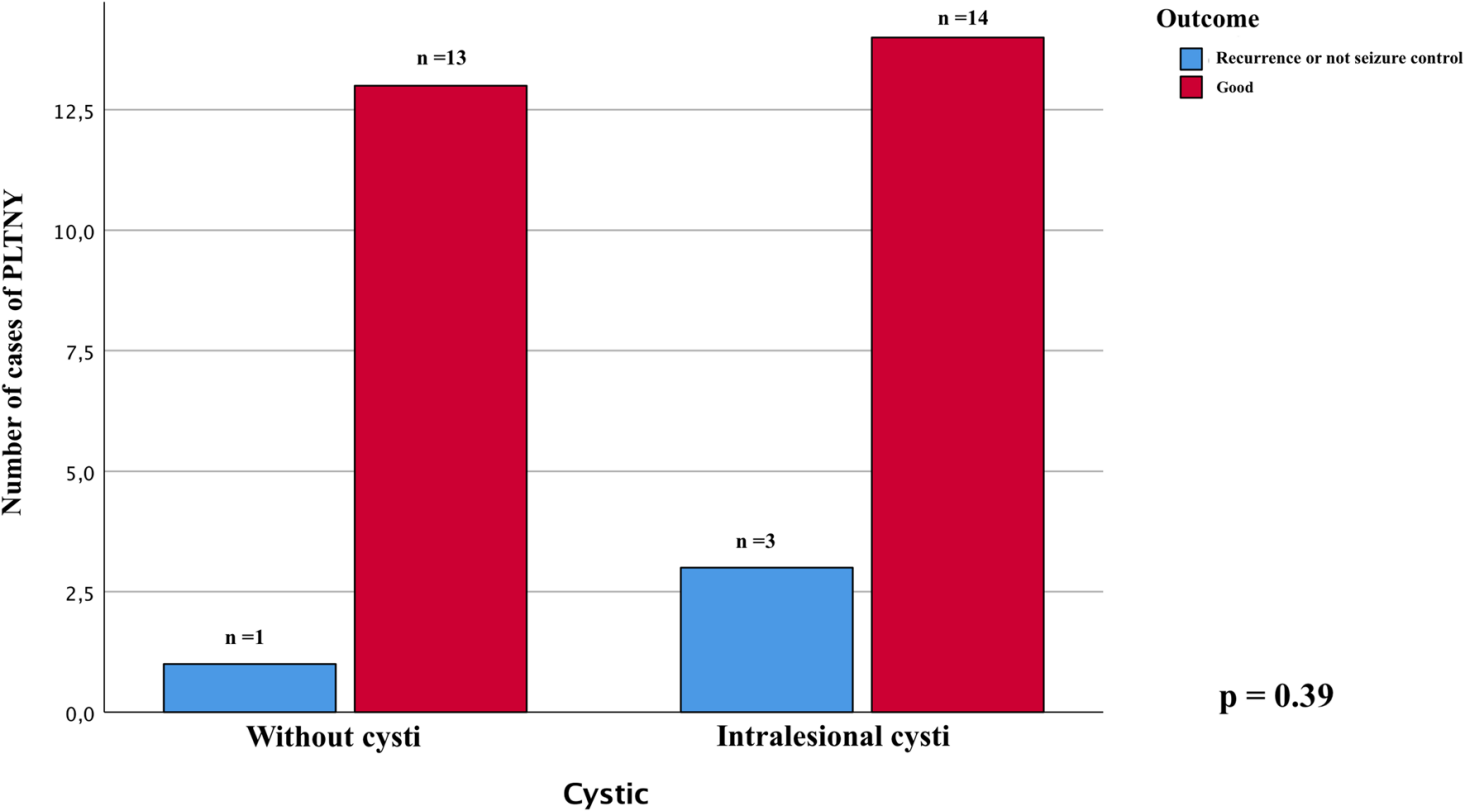
The bar chart shows that cystis is not significantly associated with treated patients' outcomes for PLTNY. Chi-square test *p* = 0.39

**Fig. 6:**
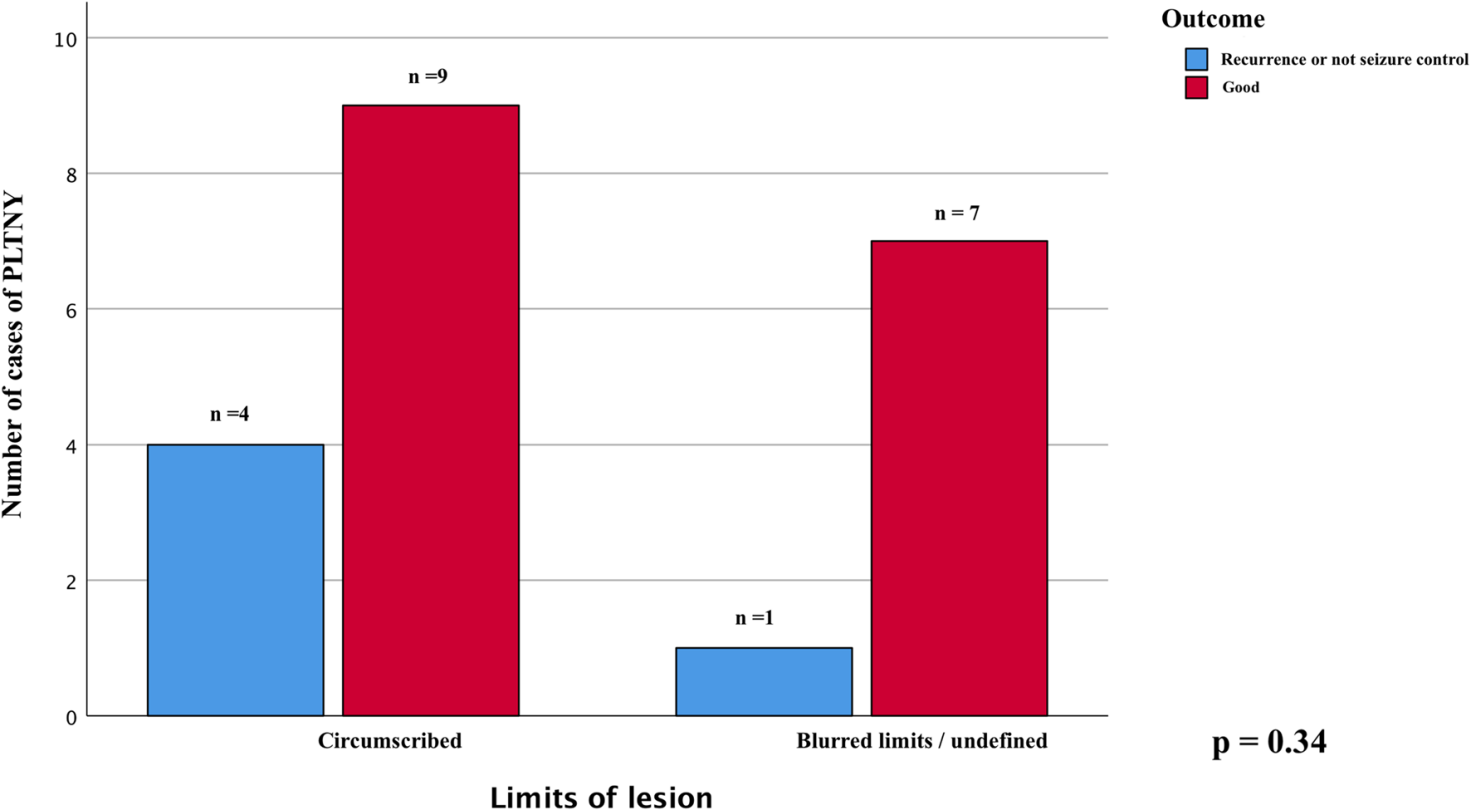
The chi-square analysis displayed graphically in this bar chart shows that the undefined limits of the lesion is not significantly associated with the outcome of treated patients (*p* = 0.34)

We compared the main radiological appearance variables reported and found no significant difference in outcome between lesions with calcifications in their context or cysts (chi-square test, *p* = 0.85 Fig. 4A and *p* = 0.39 Fig. 5, respectively). We evaluated how the presence of lesion margins defined as "blurred", i.e., tumors that are not exactly circumscribed, may impact the outcome measure, verifying that there is no statistically significant association (chi-square test, *p* = 0.34, Fig. 6).

Interesting, there is a substantial difference in outcome in cases where there is graphic contrast enhancement in the context of the tumor, where there is a more significant association of recurrence or poor control of epileptogenic symptoms (chi-square test, *p* = 0.07 Fig. 4B).

## Discussion

PLNTY was first described in 2017 [10], and today, it is part of the group of pediatric glial neoplasms of the benign type according to the WHO classification of 2021 [11, 12]. The designation of PLNTYs as “polymorphous” is meant to acknowledge that, while all harbored components prompting consideration of oligodendroglioma in the differential diagnosis, most also contained patently astrocytic or ambiguous appearance and several manifested foci of developed perivascular pseudorosetting [3].

Since its discovery, studies published on PLNTY show that predominant importance has been given to its genetic and molecular peculiarities rather than to radiological and surgical features [13]. Histologically distinguishing between the LEATs subtypes can be challenging, and molecular profiling is now recognized as critical for accurate classification [1]. Indeed, the final differential diagnosis, especially with oligodendroglioma, is often made by interpreting the histology in light of the molecular profile [1].

Characteristics shared by PLNTY and oligodendroglioma include clinical presentation and course, appearance on radiologic imaging, and histopathology. Our study shows that the age of onset is typically within the pediatric or young adult age range for the cases of PLNTY (mean 22.16) versus the peak incidence between 40 and 60 years, which is more descriptive of oligodendroglioma [5]

Both neoplasms can be incidentally found, but otherwise, patients are most likely to complain of new seizures with the epileptogenic mass [5]. The distinction of these diagnoses can be clinically significant since oligodendroglioma has more aggressive behavior than PLNTY. PLNTY is associated with an indolent course, and seizure control can typically be attained with total surgical resection [8].

Examining the outcomes of the few surgically treated patients diagnosed with PLNTY shows that the outcome is not always optimal. Indeed, even though a GTR or lobectomy occurs, the patient does not have complete control of seizures and that despite a typical genetic expression, they do not correlate with a particular outcome. Cases of recurrence or biological shift to more aggressive forms of glioma have also been identified [7]. In this study, we first argued that molecular expression might significantly affect the outcome.

PLNTY is notable for the presence of oligodendroglioma-like cellular components and CD34 immunopositivity. Immunohistochemical markers such as CD34 are other valuable tools that can readily discriminate between PLNTY and oligodendroglioma since the last staining negatively while PLNTY stains positively. Molecular analysis has identified frequent genetic abnormalities involving the B-Raf proto-oncogene (BRAF), fibroblast growth receptor (FGFR), and a distinct methylation pattern. PLNTY has been found to have a different DNA methylation signature [14], like BRAF mutation or fibroblast growth factor receptors 2 and 3 (FGFR2/FGFR3) translocation [11], involving the mitogen-activated protein kinase (MAPK) pathway [2, 3], lacking isocitrate dehydrogenase (IDH) mutations and 1p/19q codeletion (characteristic of Oligodendroglioma) [1].

The clinical significance of CD34 expression and BRAF mutation was investigated in a recent publication aiming to study the relationship between biomolecular markers and clinical–pathological features [13]. The study found that while CD34 expression was significantly associated with a longer duration of epilepsy, BRAF mutation was associated with multiple seizure types. It was suggested that BRAF-mutant protein expression might influence neural networks, causing the abnormal discharge of various populations of neurons in different locations, which in turn may contribute to the occurrence of varying seizure patterns [8].

Although the BRAF V600E genetic mutant may play a causative role in PLNTY’s growth and may be promising to pursue new medical therapy for PLNTY [5], no associations are reported with the outcome of PLNTY patients. Our study does not suggest a relationship between the expression of BRAF or FGFR mutations with the risk of poor seizure control after surgery or risk of recurrence [7]. Therefore, while molecular features better explain the initial behavior and clinic of PLNTY, we further considered in our initial hypothesis if the anatomical and radiological features of the tumor may have peculiarities in post-surgical outcome.

The most extensive published series of PLNTYs described by Huse et al. [10] showed infiltrative growth and oligodendroglioma-like histopathology. In general, the extent of the lesion is considered smaller than the other epileptogenic tumors [14]. However, this assertion is currently challenging to support, because from the cases reported in the literature, only in ten patients were tumor diameters reported. Precisely as in Huse's case series, our study confirms that most tumors localize in the temporal lobe and that the most common radiological features include cystic change, focal enhancement, and calcification [15]. Benson et al. [16] suggested that a well-circumscribed temporal lobe [17] mass with central calcifications, peripheral cysts, and the heterogeneous internal signal should have raised suspicion of a PLNTY. An oligodendroglioma may have demonstrated many of these imaging features, but the findings favored a PLNTY. Most notably, oligodendrogliomas have indistinct margins.

**Fig. 7:**
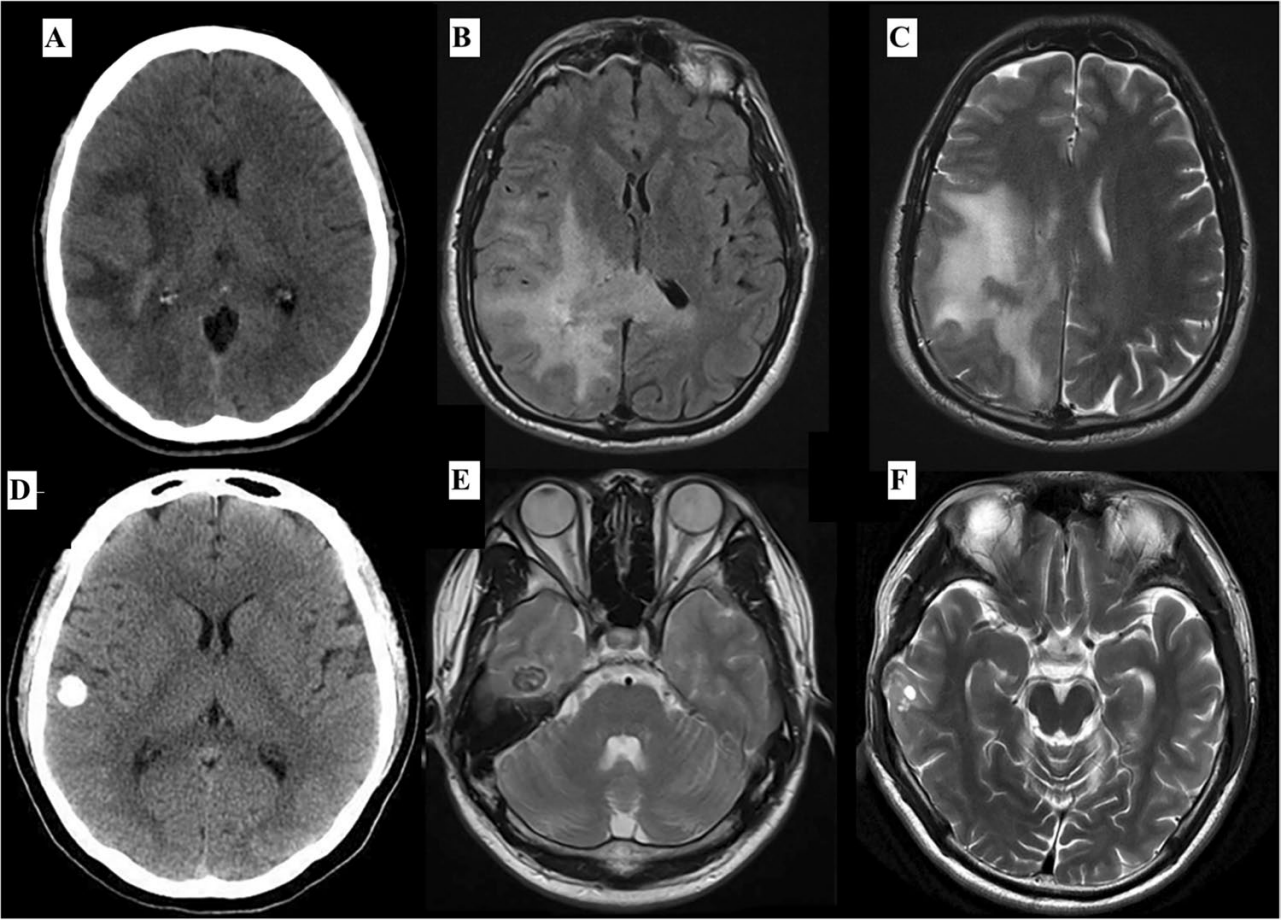
The figure shows the comparison between the radiological images of two patients with a confirmed diagnosis of PLTNY: it is show in particular the high radiological variability that can occur in CT scanning and MRI. In the first patient, a lesion without calcifications (**A**), with very blurred margins (**B**) and without cysts in context (**C**) is documented, while in the second case, a well-localized lesion in the temporal lobe (**D**) with cysts in context (**E**) with well-defined margins (**F**) is appreciated

Few PLNTY cases of the following reported articles yield insufficient knowledge of radiology to apply to the diagnostic setting. The only specific radiological study published by Chen et al. [2] demonstrated that PLNTY has a wide radiological variability, with clinical outcomes not directly dependent on the type of resection (Fig. 7). PLNTY typically appears with hyperintense or mixed density on T2 magnetic resonance imaging (MRI) and hypointense or mixed density on T1 imaging [2, 16], usually near the brain surface with frequent calcifications [17]. Calcifications are frequently seen on computed tomography (CT) images [3]. We can confirm that variable degrees of calcification are observed with a confirmed diagnosis in most cases. In a study by Suldany et al. [5], 33% demonstrated enhancement on post-gadolinium T1-weighted MR images for PLNTY. In contrast, while in the case of oligodendroglioma, contrast-enhancing is evaluated as a radiological feature of the tumor without any prognostic impact, we found that PLNTY may be correlated with poorer control of epileptic symptomatology. We suppose it may also be related to the slightly more aggressive behavior of the tumor.

The calcifications seen on MRI are also apparent in the histopathology of both tumors, and the general cell morphology of PLNTY mimics oligodendroglioma closely [5].

What is reported, however, is that in most cases, PLNTY presents as a well-circumscribed tumor [5, 17] that tends to be cystic and rich in calcifications [1], predominantly located in the temporal lobe [17] and associated with cortical dysplasia.

While all reported cases are shown as hyperintensity in T2WI and iso- or hypointensity in T1WI with slight or no enhancement after contrast-enhanced, in some cases, PLNTY tends to prominently show an infiltrative growth pattern with adjacent tissue involvement and subpial extension and partly exhibit sharp borderline [17, 18]. In contrast, other cases tend to have a completely well-defined borderline. The infiltrated growth patterns may explain the unclear boundary with normal brain tissue in radiology [19].

To better understand the characteristics of the tumor with a view to suboptimal surgical resection in our representative case, we evaluated DTI and spectroscopy values in suspicion of an early malignancy form. We identified that within a multi-voxel study, only in a deep peri-callosal portion of the tumor a mild and unremarkable choline peak was expressed compared to NAA (Fig. 1). The DTI study, on the other hand, identified white matter fiber depreciation rather than prominent infiltration, a sign that the mass effect of the lesion appears to be minimal (but present) with minor perilesional edema. This finding confirmed what was reported by Chen et al. [2], the describes a slight decrease of NAA and increase of Cho in H1-MRS, without restricted diffusion in DWI [6], and compression rather than disruption of the fiber tracts in DTI, which all suggest the PLNTY to be a low-grade, less aggressive neoplasm [2, 20].

The significant radiological variability present in the imaging of PLTNY does not only affect the different cases reported in the literature [15–20]; in fact, we argue that such variability is present macroscopically within the same lesion in the same patient, which is but also evident when the surgeon addresses the resection of the tumor mass, especially when it is poorly calcified and poorly defined in the margins, such a finding may represent an additional differential diagnostic feature concerning oligodendroglioma, as well as a further difficulty in achieving complete resection.

Further investigation will be necessary to define the clinical implications of the imaging observations described herein. In particular, it will be important to re-evaluate contrast-enhancement rates in other intra-axial pediatric and young adult brain tumors as current pathologic criteria define those entities.

## Conclusions

PLNTYs exhibit an almost invariably benign clinical course and appear well controlled by surgical resection. The prototypical PLNTY is a well-circumscribed lesion with macroscopic calcification and a cystic component located peripherally within the posteroinferior temporal lobe of a young patient. Still, from our recent analysis, its radiological and clinical variability appears to be much wider. Surgically, excision can be burdened by profound tumor variability and difficulty identifying tumor margins. The molecular driving alterations, such as BRAF V600E and FGFR translocations, do not predict the post-surgical outcome. On the radiological aspect, in PLNTY, contrast enhancement seems to impact prognosis, recurrence, and seizure control much more than radiological features of the tumor.

## Supplementary Information

Below is the link to the electronic supplementary material.

Supplementary Video 1: The video shows highlights of the surgical procedure performed in awake. After a right parietal craniotomy under neuronavigation guidance, the area of signal alteration visible on MRI was reached. Note the different moments, during intra-lesional debulking, when the change in tumor texture and color was witnessed. (MP4 30324 KB)

## Data Availability

Not applicable, at request of corresponding author (D.A.).

## Abbreviations

PLNTY: Polymorphous low-grade neuroepithelial tumor of the young
CNS-WHO: Central Nervous Tumor-World Health Organization
LEATs: Low-grade epilepsy-associated tumors
PRISMA: Preferred reporting items for systematic reviews and meta-analysis
GTR: Gross total resection
MRI: Magnetic resonance imaging
Cho-NAA: Choline/*n*-acetyl-aspartate
EOR: Extent or resection
BRAF: B-Raf proto-oncogene
FGFR: Fibroblast growth receptor
FGFR2/FGFR3: Fibroblast growth factor receptors 2 and 3
MAPK: Mitogen-activated protein kinase
IDH: Isocitrate dehydrogenase
